# Tumor suppression in skin and other tissues via cross-talk between vitamin D- and p53-signaling

**DOI:** 10.3389/fphys.2014.00166

**Published:** 2014-06-03

**Authors:** Jörg Reichrath, Sandra Reichrath, Kristina Heyne, Thomas Vogt, Klaus Roemer

**Affiliations:** ^1^Department of Dermatology, The Saarland University HospitalHomburg (Saar), Germany; ^2^José Carreras Centre and Internal Medicine I, University of Saarland Medical CentreHomburg (Saar), Germany

**Keywords:** vitamin D, vitamin D receptor, p53, MDM2, cancer

## Abstract

P53 and its family members have been implicated in the direct regulation of the vitamin D receptor (VDR). Vitamin D- and p53-signaling pathways have a significant impact on spontaneous or carcinogen-induced malignant transformation of cells, with VDR and p53 representing important tumor suppressors. VDR and the p53/p63/p73 proteins all function typically as receptors or sensors that turn into transcriptional regulators upon stimulus, with the main difference being that the nuclear VDR is activated as a transcription factor after binding its naturally occurring ligand 1,25-dihydroxyvitamin D with high affinity while the p53 family of transcription factors, mostly in the nucleoplasm, responds to a large number of alterations in cell homeostasis commonly referred to as stress. An increasing body of evidence now convincingly demonstrates a cross-talk between vitamin D- and p53-signaling that occurs at different levels, has genome-wide implications and that should be of high importance for many malignancies, including non-melanoma skin cancer. One interaction involves the ability of p53 to increase skin pigmentation via POMC derivatives including alpha-MSH and ACTH. Pigmentation protects the skin against UV-induced DNA damage and skin carcinogenesis, yet on the other hand reduces cutaneous synthesis of vitamin D. A second level of interaction may be through the ability of 1,25-dihydroxyvitamin D to increase the survival of skin cells after UV irradiation. UV irradiation-surviving cells show significant reductions in thymine dimers in the presence of 1,25-dihydroxyvitamin D that are associated with increased nuclear p53 protein expression, and significantly reduced NO products. A third level of interaction is documented by the ability of vitamin D compounds to regulate the expression of the *murine double minute 2* (*MDM2*) gene in dependence of the presence of wild-type p53. MDM2 has a well-established role as a key negative regulator of p53 activity. Finally, p53 and family members have been implicated in the direct regulation of VDR. This overview summarizes some of the implications of the cross-talk between vitamin D- and p53-signaling for carcinogenesis in the skin and other tissues.

## Skin, VDR and the vitamin D endocrine system/regulatory network: an introduction

**Figure 1:**
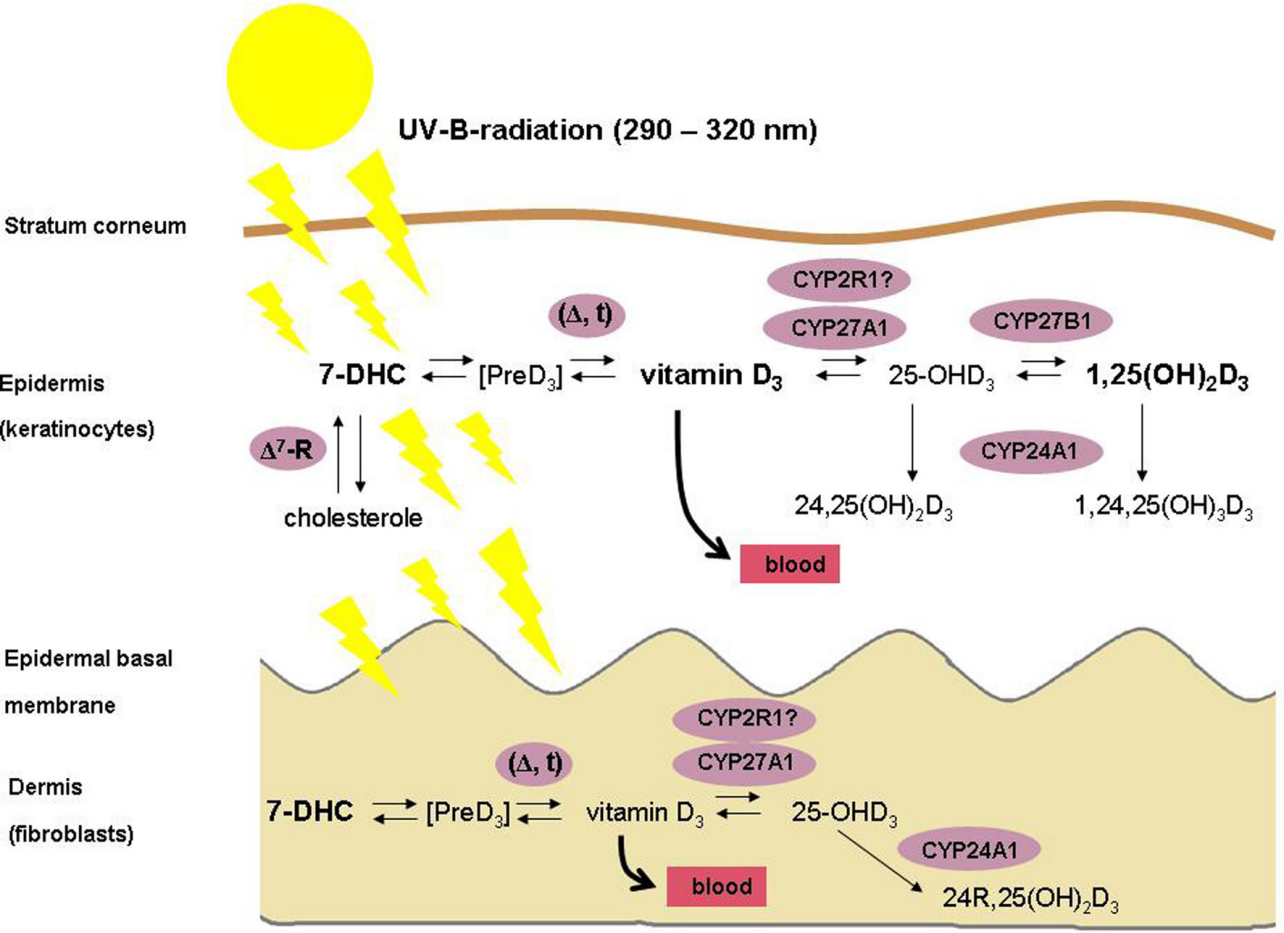
**Schematic illustration of the vitamin D metabolism in human skin**. Please note that, in contrast to fibroblasts, keratinocytes possess the enzymatic machinery for the complete synthesis of 1,25-dihydroxyvitamin D from 7-dehydrocholesterole (7-DHC).

The skin is the largest organ of the human body, consisting of several compartments that are named epidermis, dermis and subcutis. The epidermis contains a basal layer (stratum basale), that is composed of self-renewing cells (keratinocytes) with limited proliferative capacity (transient amplifying cells), of stem cells with high proliferative capacity that need to be preserved, and of outwardly migrating layers (stratum spinosum, stratum granulosum and stratum corneum) of mostly resting keratinocytes at different stages of differentiation. The skin is one of the key tissues of the human body's vitamin D regulatory network (VDRN) (Lehmann et al., 2004; Holick, 2007; Reichrath and Reichrath, 2012; Mason and Reichrath, 2013). First, vitamin D is synthesized in the skin (Figure 1) by the action of solar or artificial ultraviolet B (UVB) radiation (under most living conditions, only a small amount of vitamin D is taken up by the diet) (Lehmann et al., 2004; Holick, 2007). Second, the skin represents an important target tissue for 1,25-dihydroxyvitamin D, the biologically active natural vitamin D metabolite, that is formed from vitamin D by consecutive hydroxylations at position 25 in the liver (mediated by CYP2R1 and by CYP27A1, resulting in 25-hydroxyvitamin D) and at position 1 in the kidneys and in many other tissues (mediated by CYP27B1) (Lehmann et al., 2004; Holick, 2007; Reichrath and Reichrath, 2012; Mason and Reichrath, 2013). 1,25-Dihydroxyvitamin D represents a potent seco-steroid hormone that regulates, via various independent mechanisms growth, many non-malignant and malignant cell types, including human keratinocytes (Lehmann et al., 2004; Holick, 2007; Haussler et al., 2012; Reichrath and Reichrath, 2012; Mason and Reichrath, 2013). It exerts its effects through the binding with high affinity to a corresponding receptor (VDR) that is located intranuclear in target tissues (Lehmann et al., 2004; Holick, 2007; Haussler et al., 2012; Reichrath and Reichrath, 2012; Mason and Reichrath, 2013). VDR is a member of a superfamily named trans-acting transcriptional regulatory factors, that also contains the retinoic acid receptors (RARs) and the retinoid-X receptors (RXRs), as well as the thyroid and steroid hormone receptors (Lehmann et al., 2004; Holick, 2007; Haussler et al., 2012; Reichrath and Reichrath, 2012; Mason and Reichrath, 2013). The farnesoid-X receptor (FXR) that controls bile acid metabolism and the pregnane-X receptor (PXR) which regulates xenobiotic detoxification are evolutionarily most closely related to the VDR (Haussler et al., 2012). Binding of its ligand 1,25-dihydroxyvitamin D induces conformational changes of the VDR that lead to heterodimerization with RXR and to zinc finger-mediated binding to vitamin D response elements (VDREs) that are located in regulatory regions of target genes (Haussler et al., 2012). As a result, vitamin D activity in a particular cell largely depends upon sufficient expression of VDR and RXR proteins, the autocrine/paracrine production or the endocrine delivery of adequate amounts of the 1,25-dihydroxyvitamin D ligand, and of cell-specific programming of gene transcription to regulate expression of distinctive genes that encode proteins that finally exert the vitamin D effect (Haussler et al., 2012). cDNA microarray analyses of mRNAs and other investigations suggest that as many as 500–1000 coding genes may be regulated by the VDR, which may contact up to ~8000 loci in the human genome (Haussler et al., 2012). 1,25-Dihydroxyvitamin D-mediated transcriptional regulation of many genes involved in cellular growth and differentiation has been demonstrated, including the genes for ß_3_-integrin, fibronectin, and cell cycle regulatory proteins such as p21/WAF-1 (CDKN1A) (Lehmann et al., 2004; Holick, 2007; Haussler et al., 2012). Like most other skin cells, keratinocytes express VDR (Lehmann et al., 2004; Holick, 2007); in these cells, 1,25-dihydroxyvitamin D, blocks proliferation and promotes differentiation *in vitro* (Lehmann et al., 2004; Holick, 2007; Haussler et al., 2012). Interestingly, it has been reported that the combination of 1,25-dihydroxyvitamin D and the retinoic acid metabolite isotretinoin is efficient in the therapy of precancerous skin lesions and of non-melanoma skin cancer (cutaneous squamous and basal cell carcinomas) (Tang et al., 2012a,b; Mason and Reichrath, 2013). Moreover, it has been demonstrated that VDR ablation promotes chemically induced skin carcinogenesis (Tang et al., 2012a,b; Mason and Reichrath, 2013).

VDR-signaling comprises much more than just ligand/ receptor triggering of gene expression. Distinct and fine-tuned responses indicate a complex regulation of this signaling pathway. Moreover, chemical and other modifications of the VDR signaling pathway govern such important parameters as intracellular trafficking, duration of interaction between the receptor and cofactors, the receptor and ligand, as well as turnover and stability of other relevant proteins (Haussler et al., 2012). Not least, regulation of VDR target genes is controlled by stability and turnover of relevant microRNAs and RNAs (Haussler et al., 2012).

Depending on cell type and context, both VDR- and p53-signaling regulate many cellular functions that are of relevance for cancer development, including proliferation, differentiation, apoptosis and cell survival (Murray-Zmijewski et al., 2006; Holick, 2007; McKeon and Melino, 2007; Vousden and Lane, 2007; Vousden and Prives, 2009; Haussler et al., 2012; Mason and Reichrath, 2013). Consequently, vitamin D- and p53-signaling pathways have a significant impact on spontaneous or carcinogen-induced malignant transformation of cells, with vitamin D receptor (VDR) and p53 representing important tumor suppressors (Murray-Zmijewski et al., 2006; Holick, 2007; McKeon and Melino, 2007; Vousden and Lane, 2007; Vousden and Prives, 2009; Haussler et al., 2012; Mason and Reichrath, 2013). Mutations in genes encoding for proteins of the p53 pathway represent a hallmark of many if not all types of cancer (Vousden and Lane, 2007; Vousden and Prives, 2009). Low serum 25(OH)D concentrations and distinct polymorphisms (SNPs) in the *VDR* gene and other vitamin D-related genes, on the other hand, are associated with an increased incidence and an unfavorable outcome of various malignancies (Mason and Reichrath, 2013). The VDR and the p53 family all function typically as activatable transcriptional regulators, with the main difference being that VDR is activated after binding its naturally occurring ligand 1,25-dihydroxyvitamin D (1,25(OH)_2_D or calcitriol) with high affinity (Haussler et al., 2012) while p53, mostly in the nucleoplasm, responds to a large and still growing number of alterations in cell homeostasis (Murray-Zmijewski et al., 2006; McKeon and Melino, 2007; Vousden and Lane, 2007; Vousden and Prives, 2009). In any event is the result of such activation—manifested by conformational changes and heterodimerization with retinoid X receptor (RXR) of VDR and by chemical modifications and oligomerization of the p53 family—the direct contact with regulatory DNA. In both pathways the cell type- and context-dependent recruitment of nuclear co-regulators entails the stimulation or repression of a very large number, typically hundreds, of genes (Lin et al., 2005; Holick, 2007; Perez and Pietenpol, 2007; Sbisa et al., 2007; Riley et al., 2008; Haussler et al., 2012). Several of these code themselves for transcriptional regulators, adding a further level of complexity to the networks. It is obvious that transcription factor pathways may cross-talk, for instance, through the sharing of target genes or co-regulators, and through the engagement in interdependent regulatory loops. Indeed, all of these mechanisms, plus several others, seem to have been realized in the cross-talk of VDR and the p53 family (Table 1).

**Table 1:** **Overview of the cross talk between vitamin D- and p53 signaling**.

**Crooss talk/interaction**	**Mechanism**	**References**
p53 modulates cutaneous vitamin D synthesis	p53 upregulates skin pigmentation via POMC derivatives including alpha-MSH and ACTH.	Rev. in Yamaguchi and Hearing, 2009 (77)
p53 regulates VDR expression	p53 and its family members have been implicated in the direct regulation of the VDR.	Maruyama et al., 2006 (97)
	p53 protein binds to highly conserved intron-sequences of the VDR gene.	Kommagani et al., 2007 (96)
1,25-D increases survival of UV-irradiated skin cells	Significant reductions in thymine dimers in the presence of 1,25-D in UV-irradiated, surviving cells that are associated with increased nuclear p53 protein expression.	Gupta et al., 2007 (78)
1,25-D regulates MDM2 expression	Dependent on presence of wild type p53, 1,25-D regulates expression of the MDM2 gene.	Chen et al., 2013 (79)
	Interaction between VDRE and p53Res in the P2 promoter region of the MDM2 gene.	

1,25-D, 1,25-dihydroxyvitamin D; MDM2, murine double minute 2.

Intriguingly, both pathways are critically involved in cellular processes that are important for carcinogenesis such as cell differentiation/proliferation, in the regulation of stem cell maintenance, and in cell homeostasis. While VDR controls proliferation/differentiation of many cell types (Holick, 2007; Haussler et al., 2012), some members and isoforms of the p53 family, and in particular p53 itself, reduce the stem cell potential and stimulate differentiation (Lin et al., 2005). Interestingly, on the side of the p53 family, all three members (p53/p63/p73) can be expressed as truncated isoforms capable of counteracting their siblings' transactivating effects (Murray-Zmijewski et al., 2006). Not too surprising, VDR and p53 have been linked to many malignancies, including non-melanoma skin cancer (Mason and Reichrath, 2013). The present review aims at providing an overview on this interesting signaling network, with a focus on non-melanoma skin cancer. Future genome-wide analyses of the target genes will shed further light on the interaction of these pleiotropic regulators. Before the cross-talk is discussed, the p53 pathway shall be briefly outlined.

## The p53 family of transcriptional regulators

p53, p63, and p73 (the p53 family hereafter) are homotetrameric transcriptional regulators that bind to very closely related DNA motifs, consisting of two consecutive 10-mers (half-sites), preferentially spaced by no more than zero to 2 base pairs, with the consensus r,r,r,C,A/T,T,G,y,c,y (p53); r,r,r,C,G,T,G,y,y,y; t/a,a/t,a,C,A/T,T,G,t,t/a,t or r,r,r,C,A/G,T/A,G,y,y,y (p63), and a/c/g,g/a,g,C,A,T,G,c/t,c,c/t (p73; r = purines; y = pyrimidines) (Osada et al., 2005; Riley et al., 2008; Brandt et al., 2009; Roemer, 2012). They share a large number of target sequences, as expected given the high degree of homology within the DNA binding domains, among the consensus sequence motifs, and the degeneracy of the individual binding sites (Roemer, 2012). It is therefore perhaps no surprise that the regulation of a defined sequence by any of these transcription factors is controlled at several levels including posttranslational modifications and protein/protein interactions. Many of these are specific for each family paralog. Moreover, the binding of p53, p63, and p73 to DNA is affected by additional parameters such as the number of the half-sites, their orientation, their position relative to the target gene, and their overlap with binding sites for other transcription factors. Finally, differential recruitment of co-activator/co-repressor complexes to promoters has been documented. These may be coded, for example, by specific spacings between the 10-mers of the DNA binding motifs (Riley et al., 2008). Epigenetic CpG methylation does not seem to affect the binding to DNA significantly (Brandt et al., 2009; Roemer, 2012).

The p53 family proteins display a modular organization that is quite different from that of the VDR (see above). Typically, an N-terminal transactivation domain (TD), a central DNA binding domain (DBD) and C-terminal regulatory and protein/protein interaction domain is present. The DBDs are the most highly conserved regions among the paralogs, sharing ~60% homology (Murray-Zmijewski et al., 2006; Roemer, 2012). In addition to the full-length variants, a large number of isoforms exists, owing to transcription initiation from internal promoters, alternative splicing and the use of alternative translation initiation sites; however, in most cases the DBD is maintained. More than 10 different isoforms of p53, more than six of p63 and at least 29 of p73 are currently known (Murray-Zmijewski et al., 2006; Hollstein and Hainaut, 2010; Roemer, 2012). In most cases, their biological functions are not fully understood. Furthermore, an arsenal of posttranslational modifications that are in part interdependent has evolved. These include phosphorylations, acetylations, ubiquitinations, sumoylations, neddylations, methylations, glycosylations, and oxidation/reduction, and they control the proteins' abundance, DNA binding, level of activity as transcription factor, cross-talk with other proteins and subcellular localization (Murray-Zmijewski et al., 2006; Toledo and Wahl, 2006; Kruse and Gu, 2009). All these levels of regulation are best studied in p53 and have revealed an enormous degree of complexity (Vousden and Prives, 2009; Roemer, 2012) which may be exemplified by the chemical modification “code” that seems to regulate p53 function in a tissue-specific manner through the sequential build-up of poly-phosphorylation patterns at different sites and that may even be accompanied by other chemical changes such as acetylations (Gu and Roeder, 1997; Ashcroft et al., 2000; Wang et al., 2004; Roemer, 2012). At the level of the cell, p53 is involved in the regulation of the cell cycle (Wang and El-Deiry, 2006), cell survival and autophagy, DNA repair, respiration, oxidative stress protection, glucose metabolism, cell adhesion/motility, the cytoskeleton and endo/exosome compartments, and of angiogenesis. At the organismal level, p53 is involved in tumor suppression and maintenance of genome stability, and the control of stem cell compartments, female fertility and ageing (Riley et al., 2008; Roemer, 2012).

The complexity of the regulation of the p53 family is further highlighted by the antagonistic partnership between p53 and its central negative regulators, the E3 ubiquitin ligases murine double minute 2 (MDM2) and MDM4 (Roemer, 2012). Activation of p53 is almost always involving inhibition of MDM2/4. For example, acetylation of p53 and MDM2 overcomes the inhibitory ubiquitination of p53 by MDM2 through the blocking of MDM2 enzymatic function, the dissociation of the p53/MDM2 complex and thereby, the stimulation of p53's interaction with DNA as well as the recruitment of co-activators (Gu and Roeder, 1997; Ashcroft et al., 2000; Wang et al., 2004; Roemer, 2012). MDM4 is not functioning as a ubiquitin ligase for p53 but can inhibit p53's transcriptional activity and modulate the p53/MDM2 interaction (Toledo and Wahl, 2006; Roemer, 2012). Since p53 can transactivate the *MDM2* gene, a negative feedback loop is formed (Toledo and Wahl, 2006; Kruse and Gu, 2009; Roemer, 2012). Such a loop is also established with p63 and p73; however, MDM2 inhibits these transcription factors at promoters yet in contrast to p53 cannot ubiquitin-mark them for degradation (Murray-Zmijewski et al., 2006; Roemer, 2012).

Cell context determines the respective function of individual p53 family members. In the absence of extra stress, i.e., under physiological background stress induced, for instance, by reactive oxygen species (ROS) as a by-product of respiration, p53, p63, and p73 primarily control cell fate, differentiation and development. Intriguingly, these functions seem to be predominantly mediated by the DNA binding competent yet transactivation impaired delta-N isoforms of the proteins (ΔNp63, ΔNp73). In cells or tissue that have been challenged by further stresses, as for example by overt ROS production, radiation, hypoxia, hypo/hyperthermia, metabolite shortages and imbalances, oncogene dysregulation, and virus/bacterial/parasite infections, the p53 family members, and in particular p53 itself, seem to mainly control repair, proliferative capacity and survival. Central to these functions are the transactivation-proficient isoforms (p53, TAp63, TAp73). Since many of the damaging stresses can support cell transformation, the p53 family, and here again, mostly p53 itself, thus act as tumor suppressors by inducing cell cycle arrest, temporary or permanent senescence, apoptosis, and differentiation (Vousden and Lane, 2007; Levine and Oren, 2009; Vousden and Prives, 2009; Roemer, 2012). Conversely, lack of proper function of p53 or p73, or overproduction of dominant-negative ΔNp63, support tumor formation in animals and humans. Along the same line, tumor-inducing viruses encode proteins that target p53, and perhaps there is no tumor in which the p53 pathway itself plus all ascending/descending pathways are fully intact (Gatza et al., 2007; Vousden and Lane, 2007; Feng et al., 2008; Hu et al., 2008; Roemer, 2012).

Like p53, p63, and p73 can act as tumor suppressors, although this does not seem to be their primary functions (Murray-Zmijewski et al., 2006; Roemer, 2012). For example, p63 and p73 are not as frequently mutated in human cancers as is p53. Rather, p63 is often overproduced in tumors (Park et al., 2000), which seems to contradict its function as a tumor suppressor, yet as mentioned above, this is often due to p63 isoforms that lack the transactivation domain but not their ability to bind to DNA and that thereby may act dominant-negatively (Candi et al., 2007; Roemer, 2012). In contrast and as expected from a tumor suppressor, transactivation competent p63 (TAp63) can sensitize cells to apoptosis in response to DNA damaging stress (Gressner et al., 2005; Roemer, 2012). Moreover, some p63± mice are tumor-prone, and the resulting tumors often display loss of the remaining wild-type allele (Flores, 2007; Roemer, 2012). Mice with a specific deficiency for TAp73 show genomic instability and a higher tumor incidence (Tomasini et al., 2008; Roemer, 2012). Furthermore, p63 and p73 seem to have p53-independent roles in DNA repair (Talos et al., 2007; Lin et al., 2009; Roemer, 2012).

p63 and p73, but not p53, are crucial for embryonic development in all organisms examined so far (Danilova et al., 2008; Roemer, 2012). Although p53-deficiency interferes with mesoderm/endoderm fate determination in the frog *Xenopus* (Wallingford et al., 1997; Roemer, 2012), this condition fails to generate significant early phenotypes in mice or humans (Choi and Donehower, 1999; Varley, 2003; Roemer, 2012). However, at a more subtle level, and since p53 can induce stem cell differentiation, lack of p53 function may cause unrestrained stem cell proliferation (Gil-Perotin et al., 2006; Dumble et al., 2007; Roemer, 2012). Other more subtle functions of p53 are in mitochondrial respiration and glucose metabolism (Matoba et al., 2006; Roemer, 2012). Overactivity of p53, by contrast, does indeed entail immediate and dramatic consequences in the development of the early mouse embryo—its apoptotic loss—and one of the most striking functions of the p53 inhibitors MDM2 and MDM4 during embryonic development is the prevention of this consequence (Marine et al., 2006; Roemer, 2012). Later in embryonal development, for example during neurogenesis, the DNA binding-proficient yet transactivation-incompetent dominant-negative isoform of p73, ΔNp73, may serve as a p53 and p63 restraining factor to inhibit apoptosis (Jacobs et al., 2004; Roemer, 2012). p53 and possibly p63, but most importantly p73, help shape the nervous system during life, perhaps primarily by controlling apoptosis (Jacobs et al., 2005; Miller and Kaplan, 2007; Roemer, 2012).

p63 function during development is critical for epithelial stem cell maintenance (Yi et al., 2008; Roemer, 2012), squamous epithelial differentiation and skin renewal (Truong et al., 2006; Koster et al., 2007; Mikkola, 2007; Roemer, 2012). The ΔNp63 isoform acts mainly through controlling the expansion of epithelial layers while TAp63 seems to support differentiation, and it functions as the guardian of the female germ line by inducing apoptosis in damaged resting oocytes (Suh et al., 2006; Roemer, 2012). p73 deficiency in mice results in neuronal and olfactory dysfunctions as well as in chronic infection and inflammation (Murray-Zmijewski et al., 2006; Roemer, 2012). Collectively, the stem cell/differentiated cell decision seem to be regulated in part by the balance between the ΔNp63/TAp63 antagonists in the skin and - in an analogous manner - by the balance between the ΔNp73/TAp73 antagonists in the developing nervous and immune systems. Thus, p73 may be to neuronal development and homeostasis what p63 is to the development and homeostasis of the skin (De Laurenzi et al., 2000; Jacobs et al., 2004; Roemer, 2012). Since the pleiotropic VDR is important for tumor suppression as well as skin development and differentiation, it is perhaps no surprise that both transcriptional regulator pathways talk to each other.

## Cross-talk between the VDR and the p53 family in cancer

An increasing body of evidence points to a cross-talk between vitamin D- and p53-signaling occuring at different levels that might be of great importance for many malignancies, including non-melanoma skin cancer (Table 1). Both p53 and VDR act as tumor suppressors in several tissues, including skin. Much of the tumor suppressor function in the skin may be mediated through the interaction of the VDR and p53 pathways—either by mutual activation or inhibition. What is known about this interaction, in particular in non-melanoma skin cancer? DNA damage induced by solar or artificial ultraviolet (UV) radiation represents the most important environmental risk factor for carcinogenesis of cutaneous squamous cell carcinoma (SCC) (Reichrath and Reichrath, 2012; Mason and Reichrath, 2013). The predominant types of DNA damage which are directly induced by UV are promutagenic pyrimidine dimers (Wikonkal and Brash, 1999; Reichrath and Reichrath, 2012; Mason and Reichrath, 2013). Thymine-thymine dimers, which represent cys-syn cyclobutane pyrimidine dimers (CPDs), are the major form of pyrimidine dimers that are identified in human skin following UV-B exposure. In contrast, other types of DNA damage, including cytosine-cytosine, thymine-cytosine bipyrimidines, and 6–4 photoproducts are less frequently detected (Douki et al., 2000; Cooke et al., 2003; Courdavault et al., 2004a; Mouret et al., 2006; Reichrath and Reichrath, 2012; Mason and Reichrath, 2013). CPDs are caused via disruption of the 5–6 double bonds in two adjacent pyrimidine bases, thereby inducing atypical covalent binding which connects the 2 bases by a stable ring configuration, resulting in a bipyrimidine (Ravanat et al., 2001; Pattison and Davies, 2006; Reichrath and Reichrath, 2012; Mason and Reichrath, 2013). In general, CPDs are induced by UV-B (290–320 nm) (Reichrath and Reichrath, 2012; Mason and Reichrath, 2013), although the production of thymine dimer by UV-A (320–400 nm) wavelengths below 330 nm has also been reported (Applegate et al., 1999; Jiang et al., 1999; Rochette et al., 2003; Courdavault et al., 2004b; Mouret et al., 2006; Reichrath and Reichrath, 2012; Mason and Reichrath, 2013). UV-radiation induces gene mutations which may result in photocarcinogenesis (Hart et al., 1977; Sutherland et al., 1985; Brash et al., 1991; Agar et al., 2004; Besaratinia et al., 2008; Reichrath and Reichrath, 2012; Mason and Reichrath, 2013). Moreover, it has been shown that DNA damage initiates and promotes cellular mechanisms which block the detection and elimination of transformed cells by immune surveillance (Applegate et al., 1989; Kripke et al., 1992; Reichrath and Reichrath, 2012; Mason and Reichrath, 2013). UV radiation induces different forms of DNA lesions which are generated either photochemically and directly or indirectly by UV activation of several photoreceptors which have the capacity to modulate the cellular redox equilibrium, thereby generating reactive oxygen species (ROS) (Reichrath and Reichrath, 2012; Mason and Reichrath, 2013). ROS induced cellular damage then leads both to oxidative DNA damage, and to lipid peroxidation (Reichrath and Reichrath, 2012; Mason and Reichrath, 2013). Additionally, UV-induced increased levels of nitric oxide synthase (Deliconstantinos et al., 1995; Bruch-Gerharz et al., 1998; Cals-Grierson and Ormerod, 2004; Reichrath and Reichrath, 2012; Mason and Reichrath, 2013) cause excess levels of nitric oxide (NO) (Paunel et al., 2005; Mowbray et al., 2009; Reichrath and Reichrath, 2012; Mason and Reichrath, 2013). It has been shown that these pathophysiologically increased concentrations of NO and ROS combine to generate genotoxic NO products, including peroxynitrite, which modify the bases and the sugar-phosphate scaffold of DNA via nitrosative and oxidative damage (Reichrath and Reichrath, 2012; Mason and Reichrath, 2013) Both UV- and ROS-induced damages activate p53.

UV-irradiation induces p53 to stimulate skin pigmentation via POMC derivatives including alpha-MSH and ACTH (Yamaguchi and Hearing, 2009), thereby protecting the skin against further UV-induced DNA damage and skin carcinogenesis. However, this reduces cutaneous synthesis of vitamin D. This may be important because, on a second level, 1,25-dihydroxyvitamin D can increase the survival of UV-irradiated keratinocytes and prevent further accumulation of DNA damage in these surviving skin cells (Gupta et al., 2007). Following UVR, the survival of 1,25-dihydroxyvitamin D-treated skin cells was significantly higher as compared to vehicle-treated cells (*P* < 0.01) (Gupta et al., 2007). In that study, UVR-surviving and 1,25-dihydroxyvitamin D-treated keratinocytes showed significantly reduced levels of thymine dimers (TDs) as compared to vehicle-treated cells (*P* < 0.001) (Gupta et al., 2007). Following UVR, nuclear p53 protein levels were elevated and, notably, became elevated to significantly higher levels in the presence of 1,25-dihydroxyvitamin D (*P* < 0.01). In contrast, NO derivatives were significantly decreased in 1,25-dihydroxyvitamin D-treated keratinocytes (*P* < 0.05) (Gupta et al., 2007). Both the elevated levels of nuclear p53 protein and the decreased production of nitric oxide products were suggested to be responsible at least in part for the decrease in TDs seen with 1,25-dihydroxyvitamin D-treatment after UVR (Gupta et al., 2007). In addition, a reduction in the number of TDs (*P* < 0.05) and in sunburn cells (*P* < 0.01) were demonstrated in skin sections from Skh:hr1 mice that had been treated with 1,25-dihydroxyvitamin D, at 24 h after UVR (Gupta et al., 2007). It was concluded that the vitamin D system in skin, in combination with p53, may represent an intrinsic mechanism that protects against UV damage (Gupta et al., 2007).

As a further molecular level of interaction it has been demonstrated that vitamin D compounds can regulate the expression of the MDM2 gene in dependence of p53 (Chen et al., 2013). As outlined above, *MDM2* represents a p53-inducible gene that encodes an E3 ubiquitin ligase mainly responsible for the degradation of p53 by the 26S proteasome (Roemer, 2012). A major function of MDM2 is its role as a key negative feed-back regulator of p53 activity (Bond et al., 2004), p53 activates *MDM2* expression via binding to corresponding p53 response elements (p53REs) in the P2 promoter of the *MDM2* gene. The increase of MDM2 protein then leads to its binding to p53 (primarily at the N-terminal 1–52 residues), which causes degradation of p53 or inhibition of p53 activity as a transcription factor (Chen et al., 1993). Vitamin D may thus prevent a lasting and overt p53 response in the face of damage and may thereby protect reparable cells from p53-induced apoptosis. However, MDM2 also exerts many p53-independent functions, and interacts with a broad variety of other proteins (including insulin like growth factor receptor, androgen receptor, estrogen receptor, Numb, RB, p300, etc.) that are of importance for various cellular functions including proliferation/differentiation, cell fate determination, and signaling (Ries et al., 2000; Ganguli and Wasylyk, 2003; Steinman et al., 2004; Zhang and Zhang, 2005; Lengner et al., 2006; Araki et al., 2010). VDR may activate the *MDM2* gene directly, through a VDR-response element in the promoter P2 of the *MDM2* gene (Barak et al., 1994; Zauberman et al., 1995; Roemer, 2012; Chen et al., 2013) However, even with this direct binding to *MDM2* sequences, p53 seems to be required for the induction of *MDM2* expression by VDR (Chen et al., 2013). Perhaps this is reflecting a cross-talk between the VDR and p53 bound to DNA since the p53 response element in the *MDM2* gene is also located in promoter P2.

VDR and p53 family members act first and foremost as transcription factors, and accordingly, much of the highly complex cross-regulation between them seems to happen at this level. For example, members of the p53 family including ΔNp63 can modulate VDR signaling through competitive binding to various VDR target genes including *p21Waf1/Cip (CDKN1A)*. Multiple VDREs have recently been identified in the promoter region of the *CDKN1A* gene, which is a transcriptional target of p53 and encodes a powerful blocker of the cell cycle in G1 and G2 phases (Saramaki et al., 2006). Notably, like with the *MDM2* gene, the VDR and p53 binding sites are in close proximity in the *CDKN1A* promoter (Saramaki et al., 2006). A much more detailed and unbiased (pathway-independent) genome-wide analysis of the VDR:p53 family interactions bound to DNA is in need. To this end, it shall be useful to employ chromatin immunoprecipitations (ChIPs) with either ChIPping with p53-antibodies first and re-ChIPping with VDR antibodies, or *vice versa*. Also, knock-in studies in which VDR response elements or p53 response elements in close proximity are deleted, should provide a deeper insight into the cooperativity or antagonism between these important tumor-suppressing transcription factors.

In the skin, p53/p63 play an important regulatory role in the maintenance of the stem cells as well as in the establishment of the differentiation gradient. In the undifferentiated proliferating basal layer of the skin, the dominant negatively acting, because DNA binding but transactivation impaired, ΔNp63 rules. Most effects exerted by the transactivation-competent p53 family members are inhibited by it (Yang et al., 1998; Lee and Kimelman, 2002; Roemer, 2012) In addition, ΔNp63 may inhibit differentiation by the blunting of VDR signaling through binding to various VDR target genes including *CDKN1A* (Pellegrini et al., 2001; Westfall et al., 2003; Roemer, 2012) TAp63 that is minor to ΔNp63 in this proliferating compartment of the skin, may become more dominant as ΔNp63 levels decrease in the course of differentiation (Nylander et al., 2002; Roemer, 2012).

Finally, p53 family members may regulate VDR directly (Maruyama et al., 2006; Kommagani et al., 2007). In a comparative genomics investigation in the human and mouse genome designed to locate conserved p53 binding sites, the VDR and 31 other genes were newly described as putative p53 targets. Reverse transcription-PCR and real-time PCR confirmed the responsiveness of these genes to p53 in human cancer cell lines (Maruyama et al., 2006). It was shown that VDR is upregulated by p53 and some other members of the p53 family. For example, an isoform of p63 (p63 gamma) can specifically upregulate VDR by directly associating with the VDR promoter *in vivo* (Kommagani et al., 2007). Moreover, ChIP analysis demonstrated that wild-type p53 protein binds to a conserved intronic site of the VDR gene (Maruyama et al., 2006). Conversely, transfection of VDR into cells resulted in upregulation of several p53 target genes and in growth suppression of colorectal cancer cells. In addition and as discussed above, p53 stimulated several VDR target genes in a 1,25-dihydroxyvitamin D-dependent manner, that is, in cooperation with VDR (Maruyama et al., 2006). Future, whole transcriptome-including studies will identify new transcripts that are initiated by VDR and p53 in concert.

An increasing body of evidence highlights the relevance of the cross-talk between VDR- and p53-signaling under various physiological and pathophysiological conditions. One study identified the VDRE as overrepresented in promoter sequences bound by mutated p53 (mutp53), and showed that mutp53 can interact functionally and physically with VDR (Stambolsky et al., 2010). In that investigation, mutp53 was recruited to VDR target genes and modulated their expression (increasing transactivation or relieving repression) (Stambolsky et al., 2010). Moreover, mutp53 promoted the nuclear accumulation of VDR and converted 1,25-dihydroxyvitamin D into an anti-apoptotic agent (Stambolsky et al., 2010).

Several investigations analyzed the cross-talk between VDR- and p53-signaling in bone. It was demonstrated that hepatocyte growth factor (HGF) and 1,25-dihydroxyvitamin D act together to induce osteogenic differentiation of human bone marrow stem cells (hMSC) potentially through elevating p53 (Chen et al., 2012). The authors of this study hypothesized that the combination of HGF and 1,25-dihydroxyvitamin D can promote hMSC differentiation by up-regulation of 1,25-dihydroxyvitamin D and/or VDR expression to booster cell response(s) to 1,25-dihydroxyvitamin D. In line with this hypothesis, it was shown that HGF up-regulated gene expression of VDR and p63 and that p63 gene knockdown by siRNA eliminated the effects of HGF on VDR gene expression (Chen et al., 2012). Moreover, recent findings suggest that the cross-talk of VDR and p53 may directly target the human osteocalcin gene and positively affect osteocalcin gene expression. It was reported that osteocalcin promoter activity can be up-regulated both by exogenous and endogenous p53 and downregulated by p53-specific siRNA (Chen et al., 2011). It was shown that p53 binds to the human osteocalcin promoter *in vitro* and a p53 response element within the osteocalcin promoter region was identified (Chen et al., 2011). In this investigation, an additive effect of p53 and VDR on the regulation of osteocalcin promoter activity was observed. Another study demonstrated that p73 acts as an upstream regulator of 1,25-dihydroxyvitamin D-induced osteoblastic differentiation (Kommagani et al., 2010). In that investigation, silencing p73 significantly decreased 1,25-dihydroxyvitamin D-mediated osteoblastic differentiation; although p73 induced by DNA-damage increased 1,25-dihydroxyvitamin D-mediated differentiation of osteosarcoma cells (Kommagani et al., 2010).

## Conclusions and perspectives

VDR and the members of the p53 family are activatable transcriptional regulators that are at the hub of a common molecular network to control cell homeostasis, proliferation, differentiation and survival, and that way, act as classical tumor suppressors. Malfunction of either entails elevated susceptibility to transformation. A tissue archetypical of this interaction is the skin. Here, VDR as well as p53 and p63 control differentiation and the maintenance of the stem cell compartment. Accordingly, damage to skin cells such as induced by UV irradiation, or transformation of skin cells, typically come with characteristic responses of these proteins in the form of specific gene expression profiles to control differentiation, proliferation and survival. Since both classes of nuclear transcription factors act primarily through the regulation of genes, it is thus no great surprise to find functional interaction at several levels. Future, ChIP- and transcriptome analysis-based genome-wide studies of the DNA sequences that are targeted individually or by both factors together should provide us with new insights into this fascinating network.

### Conflict of interest statement

The authors declare that the research was conducted in the absence of any commercial or financial relationships that could be construed as a potential conflict of interest.

## References

[B1] Agar N. S. Halliday G. M. Barnetson R. S. Ananthaswamy H. N. Wheeler M. Jones A. M. (2004). The basal layer in human squamous tumours harbors more UVA than UVB fingerprint mutations: a role for UVA in human skin carcinogenesis. Proc. Natl. Acad. Sci. U.S.A. 101, 4954–4959 10.1073/pnas.0401141101 15041750 PMC387355

[B2] Applegate L. A. Ley R. D. Alcalay J. Kripke M. L. (1989). Identification of the molecular target for the suppression of contact hypersensitivity by ultraviolet radiation. J. Exp. Med. 170, 1117–1131 10.1084/jem.170.4.1117 2529340 PMC2189477

[B3] Applegate L. A. Scaletta C. Panizzon R. Niggli H. Frenk E. (1999). *In vivo* induction of pyrimidine dimers in human skin by UVA radiation: initiation of cell damage and/or intercellular communication? Int. J. Mol. Med. 3, 467–472 10.3892/ijmm.3.5.467 10202176

[B4] Araki S. Eitel J. A. Batuello C. N. Bijangi-Vishehsaraei K. Xie X. J. Danielpour D. (2010). TGF-beta1-induced expression of human Mdm2 correlates with late-stage metastatic breast cancer. J. Clin. Invest. 120, 290–302 10.1172/JCI39194 19955655 PMC2798681

[B5] Ashcroft M. Taya Y. Vousden K. H. (2000). Stress signals utilize multiple pathways to stabilize p53. Mol. Cell. Biol. 20, 3224–3233 10.1128/MCB.20.9.3224-3233.2000 10757806 PMC85616

[B6] Barak Y. Gottlieb E. Juven-Gershon T. Oren M. (1994). Regulation of mdm2 expression by p53: alternative promoters produce transcripts with nonidentical translation potential. Genes Dev. 8, 1739–1749 10.1101/gad.8.15.1739 7958853

[B7] Besaratinia A. Kim S. I. Pfeifer G. P. (2008). Rapid repair of UVA-induced oxidized purines and persistence of UVB-induced dipyrimidine lesions determine the mutagenicity of sunlight in mouse cells. FASEB J. 22, 2379–2392 10.1096/fj.07-105437 18326785 PMC2714223

[B8] Bond G. L. Hu W. Bond E. E. Robins H. Lutzker S. G. Arva N. C. (2004). A single nucleotide polymorphism in the MDM2 promoter attenuates the p53 tumour suppressor pathway and accelerates tumour formation in humans. Cell 119, 591–602 10.1016/j.cell.2004.11.022 15550242

[B9] Brandt T. Petrovich M. Joerger A. C. Veprintsev D. B. (2009). Conservation of DNA-binding specificity and oligomerisation properties within the p53 family. BMC Genomics 10:628 10.1186/1471-2164-10-628 20030809 PMC2807882

[B10] Brash D. E. Rudolph J. A. Simon J. A. Lin A. McKenna G. J. Baden H. P. (1991). A role for sunlight in skin cancer: UV-induced p53 mutations in squamous cell carcinoma. Proc. Natl. Acad. Sci. U.S.A. 88, 10124–10128 10.1073/pnas.88.22.10124 1946433 PMC52880

[B11] Bruch-Gerharz D. Ruzicka T. Kolb-Bachofen V. (1998). Nitric oxide in human skin: current status and future prospects. J. Invest. Dermatol. 110, 1–7 10.1046/j.1523-1747.1998.00084.x 9424078

[B12] Cals-Grierson M. M. Ormerod A. D. (2004). Nitric oxide function in the skin. Nitric Oxide 10, 179–193 10.1016/j.niox.2004.04.005 15275864

[B13] Candi E. Dinsdale D. Rufini A. Salomoni P. Knight R. A. Mueller M. (2007). TAp63 and DeltaNp63 in cancer and epidermal development. Cell Cycle 6, 274–285 10.4161/cc.6.3.3797 17264681

[B14] Chen H. Hays E. Liboon J. Neely C. Kolman K. Chandar N. (2011). Osteocalcin gene expression is regulated by wild-type p53. Calcif. Tissue Int. 89, 411–418 10.1007/s00223-011-9533-x 21964930

[B15] Chen H. Reed G. Guardia J. Lakhan S. Couture O. Hays E. (2013). Vitamin D directly regulates Mdm2 gene expression in osteoblasts. Biochem. Biophys. Res. Commun. 430, 370–374 10.1016/j.bbrc.2012.11.003 23149414 PMC3544976

[B16] Chen J. Marechal V. Levine A. J. (1993). Mapping of the p53 and mdm-2 interaction domains. Mol. Cell. Biol. 13, 4107–4114 7686617 10.1128/mcb.13.7.4107-4114.1993 PMC359960

[B17] Chen K. Aenlle K. K. Curtis K. M. Roos B. A. Howard G. A. (2012). Hepatocyte growth factor (HGF) and 1,25-dihydroxyvitamin D together stimulate human bone marrow-derived stem cells toward the osteogenic phenotype by HGF-induced up-regulation of VDR. Bone 51, 69–77 10.1016/j.bone.2012.04.002 22521434

[B18] Choi J. Donehower L. A. (1999). p53 in embryonic development: maintaining a fine balance. Cell. Mol. Life Sci. 55, 38–47 10.1007/s000180050268 10065150 PMC11146796

[B19] Cooke M. S. Podmore I. D. Mistry N. Evans M. D. Herbert K. E. Griffiths H. R. (2003). Immunochemical detection of UV-induced DNA damage and repair. J Immunol Methods. 280, 125–133 10.1016/S0022-1759(03)00269-2 12972193

[B20] Courdavault S. Baudouin C. Charveron M. Favier A. Cadet J. Douki T. (2004a). Larger yield of cyclobutane dimers than 8-oxo-7,8-dihydroguanine in the DNA of UVA-irradiated human skin cells. Mutat. Res. 556, 135–142 10.1016/j.mrfmmm.2004.07.011 15491641

[B21] Courdavault S. Baudouin C. Sauvaigo S. Mouret S. Candéias S. Charveron M. (2004b). Unrepaired cyclobutane pyrimidine dimers do not prevent proliferation of UV-B-irradiated cultured human fibroblasts. Photochem. Photobiol. 79, 145–151 10.1562/0031-8655(2004)079<0145:UCPDDN>2.0.CO;2 15068027

[B22] Danilova N. Sakamoto K. M. Lin S. (2008). p53 family in development. Mech. Dev. 125, 919–931 10.1016/j.mod.2008.09.003 18835440

[B23] De Laurenzi V. Raschella G. Barcaroli D. Annicchiarico-Petruzzelli M. Ranalli M, Catani M. V. (2000). Induction of neuronal differentiation by p73 in a neuroblastoma cell line. J. Biol. Chem. 275, 15226–15231 10.1074/jbc.275.20.15226 10809758

[B24] Deliconstantinos G. Villiotou V. Stravrides J. C. (1995). Release by ultraviolet B (u.v.B) radiation of nitric oxide (NO) from human keratinocytes: a potential role for nitric oxide in erythema production. Br. J. Pharmacol. 114, 1257–1265 10.1111/j.1476-5381.1995.tb13341.x 7620717 PMC1510336

[B25] Douki T. Court M. Sauvaigo S. Odin F. Cadet J. (2000). Formation of the main UV-induced thymine dimeric lesions within isolated and cellular DNA as measured by high performance liquid chromatography-tandem mass spectrometry. J. Biol. Chem. 275, 11678–11685 10.1074/jbc.275.16.11678 10766787

[B26] Dumble M. Moore L. Chambers S. M. Geiger H. Van Zant G. Goodell M. A. (2007). The impact of altered p53 dosage on hematopoietic stem cell dynamics during aging. Blood 109, 1736–1742 10.1182/blood-2006-03-010413 17032926 PMC1794064

[B27] Feng Z. Hu W. Rajagopal G. Levine A. J. (2008). The tumour suppressor p53: cancer and aging. Cell Cycle 7, 842–847 10.4161/cc.7.7.5657 18414039

[B28] Flores E. R. (2007). The roles of p63 in cancer. Cell Cycle 6, 300–304 10.4161/cc.6.3.3793 17264676

[B29] Ganguli G. Wasylyk B. (2003). p53-independent functions of MDM2. Mol. Cancer Res. 1, 1027–1035 14707286

[B30] Gatza C. Moore L. Dumble M. Donehower L. A. (2007). Tumour suppressor dosage regulates stem cell dynamics during aging. Cell Cycle 6, 52–55 10.4161/cc.6.1.3667 17245110

[B31] Gil-Perotin S. Marin-Husstege M. Li J. Soriano-Navarro M. Zindy F. Roussel M. F. (2006). Loss of p53 induces changes in the behavior of subventricular zone cells: implication for the genesis of glial tumours. J. Neurosci. 26, 1107–1116 10.1523/JNEUROSCI.3970-05.2006 16436596 PMC6674560

[B32] Gressner O. Schilling T. Lorenz K. Schulze Schleithoff E. Koch A. Schulze-Bergkamen H. (2005). TAp63alpha induces apoptosis by activating signaling via death receptors and mitochondria. EMBO J. 24, 2458–2471 10.1038/sj.emboj.7600708 15944736 PMC1173149

[B33] Gu W. Roeder R. G. (1997). Activation of p53 sequence-specific DNA binding by acetylation of the p53 C-terminal domain. Cell 90, 595–606 10.1016/S0092-8674(00)80521-8 9288740

[B34] Gupta R. Dixon K. M. Deo S. S. Holliday C. J. Slater M. Halliday G. M. (2007). Photoprotection by 1,25 dihydroxyvitamin D3 is associated with an increase in p53 and a decrease in nitric oxide products. J. Invest. Dermatol. 27, 707–715 10.1038/sj.jid.5700597 17170736

[B35] Hart R. W. Setlow R. B. Woodhead A. D. (1977). Evidence that pyrimidine dimers in DNA can give rise to tumours. Proc. Natl. Acad. Sci. U.S.A. 74, 5574–5578 10.1073/pnas.74.12.5574 271984 PMC431814

[B36] Haussler M. R. Whitfield G. K. Kaneko I. Haussler C. A. Hsieh D. Hsieh J. C. (2012). Molecular mechanisms of vitamin D action. Calcif. Tissue Int. 92, 77–98 10.1007/s00223-012-9619-0 22782502

[B37] Holick M. F. (2007). Vitamin D deficiency. N. Engl. J. Med. 357, 266–281 10.1056/NEJMra070553 17634462

[B38] Hollstein M. Hainaut P. (2010). Massively regulated genes: the example of TP53. J. Pathol. 220, 164–173 10.1002/path.2637 19918835

[B39] Hu W. Feng Z. Atwal G. S. Levine A. J. (2008). p53: a new player in reproduction. Cell Cycle 7, 848–852 10.4161/cc.7.7.5658 18414047

[B40] Jacobs W. B. Govoni G. Ho D. Atwal J. K. Barnabe-Heider F. Keyes W. M. (2005). p63 is an essential proapoptotic protein during neural development. Neuron. 48, 743–756 10.1016/j.neuron.2005.10.027 16337913

[B41] Jacobs W. B. Walsh G. S. Miller F. D. (2004). Neuronal survival and p73/p63/p53: a family affair. Neuroscientist 10, 443–455 10.1177/1073858404263456 15359011

[B42] Jiang W. Ananthaswamy H. N. Muller H. K. Kripke M. L. (1999). p53 protects against skin cancer induction by UV-B radiation. Oncogene 18, 4247–4253 10.1038/sj.onc.1202789 10435637

[B43] Kommagani R. Payal V. Kadakia M. P. (2007). Differential regulation of vitamin D receptor (VDR) by the p53 Family: p73-dependent induction of VDR upon DNA damage. J. Biol. Chem. 282, 29847–29854 10.1074/jbc.M703641200 17716971 PMC2771332

[B44] Kommagani R. Whitlatch A. Leonard M. K. Kadakia M. P. (2010). p73 is essential for vitamin D-mediated osteoblastic differentiation. Cell Death Differ. 17, 398–407 10.1038/cdd.2009.135 19779497 PMC2822032

[B45] Koster M. I. Dai D. Roop D. R. (2007). Conflicting roles for p63 in skin development and carcinogenesis. Cell Cycle 6, 269–273 10.4161/cc.6.3.3792 17224652

[B46] Kripke M. L. Cox P. A. Alas L. G. Yarosh D. B. (1992). Pyrimidine dimers in DNA initiate systemic immunosuppression in UV-irradiated mice. Proc. Natl. Acad. Sci. U.S.A. 89, 7516–7520 10.1073/pnas.89.16.7516 1502162 PMC49741

[B47] Kruse J. P. Gu W. (2009). Modes of p53 regulation. Cell 137, 609–622 10.1016/j.cell.2009.04.050 19450511 PMC3737742

[B48] Lee H. Kimelman D. (2002). A dominant-negative form of p63 is required for epidermal proliferation in zebrafish. Dev. Cell. 2, 607–616 10.1016/S1534-5807(02)00166-1 12015968

[B49] Lehmann B. Querings K. Reichrath J. (2004). Vitamin D and skin: new aspects for dermatology. Exp. Dermatol. 13, 11–15 10.1111/j.1600-0625.2004.00257.x 15507106

[B50] Lengner C. J. Steinman H. A. Gagnon J. Smith T. W. Henderson J. E. Kream B. E. (2006). Osteoblast differentiation and skeletal development are regulated by Mdm2-p53 signaling. J. Cell Biol. 172, 909–921 10.1083/jcb.200508130 16533949 PMC2063734

[B51] Levine A. J. Oren M. (2009). The first 30 years of p53: growing ever more complex. Nat. Rev. Cancer. 9, 749–758 10.1038/nrc2723 19776744 PMC2771725

[B52] Lin T. Chao C. Saito S. Mazur S. J. Murphy M. E. Appella E. (2005). p53 induces differentiation of mouse embryonic stem cells by suppressing Nanog expression. Nat. Cell Biol. 7, 165–171 10.1038/ncb1211 15619621

[B53] Lin Y. L. Sengupta S. Gurdziel K. Bell G. W. Jacks T. Flores E. R. (2009). p63 and p73 transcriptionally regulate genes involved in DNA repair. PLoS Genet. 5:e1000680 10.1371/journal.pgen.1000680 19816568 PMC2752189

[B54] Marine J. C. Francoz S. Maetens M. Wahl G. Toledo F. Lozano G. (2006). Keeping p53 in check: essential and synergistic functions of Mdm2 and Mdm4. Cell Death Differ. 13, 927–934 10.1038/sj.cdd.4401912 16543935

[B55] Maruyama R. Aoki F. Toyota M. Sasaki Y. Akashi H. Mita H. (2006). Comparative genome analysis identifies the vitamin D receptor gene as a direct target of p53-mediated transcriptional activation. Cancer Res. 66, 4574–4583 10.1158/0008-5472.CAN-05-2562 16651407

[B56] Mason R. S. Reichrath J. (2013). Sunlight vitamin D and skin cancer. Anticancer Agents Med. Chem. 13, 83–97 10.2174/187152013804487272 23094924

[B57] Matoba S. Kang J. G. Patino W. D. Wragg A. Boehm M. Gavrilova O. (2006). p53 regulates mitochondrial respiration. Science 312, 1650–1653 10.1126/science.1126863 16728594

[B58] McKeon F. Melino G. (2007). Fog of war: the emerging p53 family. Cell Cycle 6, 229–232 10.4161/cc.6.3.3876 17297295

[B59] Mikkola M. L. (2007). p63 in skin appendage development. Cell Cycle 6, 285–290 10.4161/cc.6.3.3798 17264678

[B60] Miller F. D. Kaplan D. R. (2007). To die or not to die: neurons and p63. Cell Cycle 6, 312–317 10.4161/cc.6.3.3795 17264677

[B61] Mouret S. Baudouin C. Charveron M. Favier A. Cadet J. Douki T. (2006). Cyclobutane pyrimidine dimers are predominant DNA lesions in whole human skin exposed to UVA radiation. Proc. Natl. Acad. Sci. U.S.A. 103, 13765–13770 10.1073/pnas.0604213103 16954188 PMC1564232

[B62] Mowbray M. McLintock S. Weerakoon R. Lomatschinsky N. Jones S. Rossi A. G. (2009). Enzyme-independent NO stores in human skin: quantification and influence of UV radiation. J. Invest. Dermatol. 129, 834–842 10.1038/jid.2008.296 18818674

[B63] Murray-Zmijewski F. Lane D. P. Bourdon J. C. (2006). p53/p63/p73 isoforms: an orchestra of isoforms to harmonise cell differentiation and response to stress. Cell Death Differ. 13, 962–972 10.1038/sj.cdd.4401914 16601753

[B64] Nylander K. Vojtesek B. Nenutil R. Lindgren B. Roos G. Zhanxiang W. (2002). Differential expression of p63 isoforms in normal tissues and neoplastic cells. J. Pathol. 198, 417–427 10.1002/path.1231 12434410

[B65] Osada M. Park H. L. Nagakawa Y. Yamashita K. Fomenkov A. Kim M. S. (2005). Differential recognition of response elements determines target gene specificity for p53 and p63. Mol. Cell. Biol. 25, 6077–6089 10.1128/MCB.25.14.6077-6089.2005 15988020 PMC1168821

[B66] Park B. J. Lee S. J. Kim J. I. Lee S. J. Lee C. H. Chang S. G. (2000). Frequent alteration of p63 expression in human primary bladder carcinomas. Cancer Res. 60, 3370–3374 10910040

[B67] Pattison D. I. Davies M. J. (2006). Actions of ultraviolet light on cellular structures. EXS 96, 131–157 10.1007/3-7643-7378-4_6 16383017

[B68] Paunel A. Dejam A. Thelen S. Kirsch M. Horstjann M. Gharini P. (2005). UVA induces immediate and enzyme-independent nitric oxide formation in healthy human skin leading to NO-specific signalling. J. Invest. Dermatol. 125:A3

[B69] Pellegrini G. Dellambra E. Golisano O. Martinelli E. Fantozzi I. Bondanza S. (2001). p63 identifies keratinocyte stem cells. Proc. Natl. Acad. Sci. U.S.A. 98, 3156–3161 10.1073/pnas.061032098 11248048 PMC30623

[B70] Perez C. A. Pietenpol J. A. (2007). Transcriptional programs regulated by p63 in normal epithelium and tumours. Cell Cycle 6, 246–254 10.4161/cc.6.3.3801 17297308

[B71] Ravanat J. L. Douki T. Cadet J. (2001). Direct and indirect effects of UV radiation on DNA and its components. J. Photochem. Photobiol. B. 63, 88–102 10.1016/S1011-1344(01)00206-8 11684456

[B72] Reichrath J. Reichrath S. (2012). Hope and challenge: the importance of ultraviolet (UV) radiation for cutaneous vitamin D synthesis and skin cancer. Scand. J. Clin. Lab. Invest. Suppl. 243, 112–119 10.3109/00365513.2012.682876 22536771

[B73] Ries S. Biederer C. Woods D. Shifman O. Shirasawa S. Sasazuki T. (2000). Opposing effects of Ras on p53: transcriptional activation of mdm2 and induction of p19ARF. Cell 103, 321–330 10.1016/S0092-8674(00)00123-9 11057904

[B74] Riley T. Sontag E. Chen P. Levine A. (2008). Transcriptional control of human p53-regulated genes. Nat. Rev. Mol. Cell Biol. 9, 402–412 10.1038/nrm2395 18431400

[B75] Rochette P. J. Therrien J. Drouin R. Perdiz D. Bastien N. Drobetsky E. A. (2003). UVA-induced cyclobutane pyrimidine dimers form predominantly at thymine-thymine dipyrimidines and correlate with the mutation spectrum in rodent cells. Nucleic Acids Res. 31, 2786–2794 10.1093/nar/gkg402 12771205 PMC156735

[B76] Roemer K. (2012). Notch and the p53 clan of transcription factors. Adv. Exp. Med. Biol. 727, 223–240 10.1007/978-1-4614-0899-4_17 22399351

[B77] Saramaki A. Banwell C. M. Campbell M. J. Carlberg C. (2006). Regulation of the human p21(waf1/cip1) gene promoter via multiple binding sites for p53 and the vitamin D3 receptor. Nucleic Acids Res. 34, 543–554 10.1093/nar/gkj460 16434701 PMC1351372

[B78] Sbisa E. Catalano D. Grillo G. Licciulli F. Turi A. Liuni S. (2007). p53FamTaG: a database resource of human p53, p63 and p73 direct target genes combining *in silico* prediction and microarray data. BMC Bioinformatics 8 Suppl. 1:S20 10.1186/1471-2105-8-S1-S20 17430565 PMC1885850

[B79] Stambolsky P. Tabach Y. Fontemaggi G. Weisz L. Maor-Aloni R. Siegfried Z. (2010). Modulation of the vitamin D3 response by cancer-associated mutant p53. Cancer Cell. 17, 273–285 10.1016/j.ccr.2009.11.025 20227041 PMC2882298

[B80] Steinman H. A. Burstein E. Lengner C. Gosselin J. Pihan G. Duckett C. S. (2004). An alternative splice form of Mdm2 induces p53-independent cell growth and tumourigenesis. J. Biol. Chem. 279, 4877–4886 10.1074/jbc.M305966200 14612455

[B81] Suh E. K. Yang A. Kettenbach A. Bamberger C. Michaelis A. H. Zhu Z. (2006). p63 protects the female germ line during meiotic arrest. Nature 444, 624–628 10.1038/nature05337 17122775

[B82] Sutherland B. M. Blackett A. D. Feng N. I. Freeman S. E. Ogut E. S. Gange R. W. (1985). Photoreactivation and other ultraviolet/visible light effects on DNA in human skin. Ann. N.Y. Acad. Sci. 453, 73–79 10.1111/j.1749-6632.1985.tb11799.x 3865598

[B83] Talos F. Nemajerova A. Flores E. R. Petrenko O. Moll U. M. (2007). p73 suppresses polyploidy and aneuploidy in the absence of functional p53. Mol. Cell. 27, 647–659 10.1016/j.molcel.2007.06.036 17707235

[B84] Tang J. Y. Fu T. Lau C. Oh D. H. Bikle D. D. Asgari M. M. (2012a). Vitamin D in cutaneous carcinogenesis: Part I. J. Am. Acad. Dermatol. 67, 803.e1–803.e12 10.1016/j.jaad.2012.05.044 23062903 PMC3688468

[B85] Tang J. Y. Fu T. Lau C. Oh D. H. Bikle D. D. Asgari M. M. (2012b). Vitamin D in cutaneous carcinogenesis: Part II. J. Am. Acad. Dermatol. 67, 817.e1–817.e11 10.1016/j.jaad.2012.07.022 23062904 PMC3706259

[B86] Toledo F. Wahl G. M. (2006). Regulating the p53 pathway: *in vitro* hypotheses, *in vivo* veritas. Nat. Rev. Cancer. 6, 909–923 10.1038/nrc2012 17128209

[B87] Tomasini R. Tsuchihara K. Wilhelm M. Fujitani M. Rufini A. Cheung C. C. (2008). TAp73 knockout shows genomic instability with infertility and tumour suppressor functions. Genes Dev. 22, 2677–2691 10.1101/gad.1695308 18805989 PMC2559903

[B88] Truong A. B. Kretz M. Ridky T. W. Kimmel R. Khavari P. A. (2006). p63 regulates proliferation and differentiation of developmentally mature keratinocytes. Genes Dev. 20, 3185–3197 10.1101/gad.1463206 17114587 PMC1635152

[B89] Varley J. M. (2003). Germline TP53 mutations and Li-Fraumeni syndrome. Hum. Mutat. 21, 313–320 10.1002/humu.10185 12619118

[B90] Vousden K. H. Lane D. P. (2007). p53 in health and disease. Nat. Rev. Mol. Cell Biol. 8, 275–283 10.1038/nrm2147 17380161

[B91] Vousden K. H. Prives C. (2009). Blinded by the light: the growing complexity of p53. Cell. 137, 413–431 10.1016/j.cell.2009.04.037 19410540

[B92] Wallingford J. B. Seufert D. W. Virta V. C. Vize P. D. (1997). p53 activity is essential for normal development in Xenopus. Curr. Biol. 7, 747–757 10.1016/S0960-9822(06)00333-2 9368757

[B93] Wang S. El-Deiry W. S. (2006). p73 or p53 directly regulates human p53 transcription to maintain cell cycle checkpoints. Cancer Res. 66, 6982–6989 10.1158/0008-5472.CAN-06-0511 16849542

[B94] Wang X. Taplick J. Geva N. Oren M. (2004). Inhibition of p53 degradation by Mdm2 acetylation. FEBS Lett. 561, 195–201 10.1016/S0014-5793(04)00168-1 15013777

[B95] Westfall M. D. Mays D. J. Sniezek J. C. Pietenpol J. A. (2003). The Delta Np63 alpha phosphoprotein binds the p21 and 14-3-3 sigma promoters *in vivo* and has transcriptional repressor activity that is reduced by Hay-Wells syndrome-derived mutations. Mol. Cell. Biol. 23, 2264–2276 10.1128/MCB.23.7.2264-2276.2003 12640112 PMC150720

[B96] Wikonkal N. M. Brash D. E. (1999). Ultraviolet radiation induced signature mutations in photocarcinogenesis. J. Invest. Dermatol. Symp. Proc. 4, 6–10 10.1038/sj.jidsp.5640173 10537000

[B97] Yamaguchi Y. Hearing V. J. (2009). Physiological factors that regulate skin pigmentation. Biofactors 35, 193–199 10.1002/biof.29 19449448 PMC2793097

[B98] Yang A. Kaghad M. Wang Y. Gillett E. Fleming M. D. Dötsch V. (1998). p63, a p53 homolog at 3q27-29, encodes multiple products with transactivating, death-inducing, and dominant-negative activities. Mol. Cell. 2, 305–316 10.1016/S1097-2765(00)80275-0 9774969

[B99] Yi R. Poy M. N. Stoffel M. Fuchs E. (2008). A skin microRNA promotes differentiation by repressing “stemness.” Nature 452, 225–229 10.1038/nature06642 18311128 PMC4346711

[B100] Zauberman A. Flusberg D. Haupt Y. Barak Y. Oren M. (1995). A functional p53-responsive intronic promoter is contained within the human mdm2 gene. Nucleic Acids Res. 23, 2584–2592 10.1093/nar/23.14.2584 7651818 PMC307078

[B101] Zhang Z. Zhang R. (2005). p53-independent activities of MDM2 and their relevance to cancer therapy. Curr. Cancer Drug Targets. 5, 9–20 10.2174/1568009053332618 15720185

